# Managing stress during the coronavirus disease 2019 pandemic and beyond: Reappraisal and mindset approaches

**DOI:** 10.1002/smi.2969

**Published:** 2020-07-14

**Authors:** Martin S. Hagger, Jacob J. Keech, Kyra Hamilton

**Affiliations:** ^1^ Department of Psychological Sciences University of California Merced Merced California USA; ^2^ Faculty of Sport and Health Sciences University of Jyväskylä Jyväskylä Finland; ^3^ Menzies Health Institute Queensland Griffith University Gold Coast Queensland Australia; ^4^ School of Social Sciences University of the Sunshine Coast Sippy Downs Queensland Australia; ^5^ School of Applied Psychology Griffith University Mt Gravatt Queensland Australia

**Keywords:** COVID‐19 pandemic, intervention, reappraisal, stress appraisal, stress mindset

## 1 STRESS, HEALTH, AND THE CORONAVIRUS DISEASE 2019 PANDEMIC

The novel coronavirus disease 2019 (COVID‐19) pandemic is a global public health crisis of a scale not previously experienced in modern times (Kickbusch et al., 2020). Governmental ‘lockdown’ measures aimed at minimizing virus transmission including ‘stay at home’ orders, closure of businesses and places of congregation, and travel restrictions have had a substantive societal impact that permeates almost every facet of daily life (Gostin & Wiley, 2020; Shanafelt, Ripp, & Trockel, 2020). These widespread changes represent considerable sources of stress in the population and will have deleterious effects on mental and physical health going forward. As nations begin to emerge from ‘lockdown’, the collateral damage to human health caused by these restrictions has taken centre stage, and mental health issues, particularly stress‐related conditions and outcomes, are prominent among them. The imperative for strategies to assist in managing stress and minimizing concomitant health problems has become a priority. In this commentary, we outline how stress reappraisal interventions, which have come to the fore in recent years, may be a potentially efficacious, cost‐effective way to manage stress during and post‐pandemic, and minimize the health consequences.

The health threat posed by the spread of Severe Acute Respiratory Syndrome Coronavirus 2 (SARS‐CoV‐2), the virus that causes COVID‐19, and concerns about its effects on family, friends, and colleagues, represents a substantive source of stress itself (Hamel et al., 2020; Nelson et al., 2020). Beyond this, the social effects of the lockdown measures such as concerns over availability of food and household goods and social isolation also present as important stressors (S. K. Brooks et al., 2020; Garfin, Silver, & Holman, 2020; Hamel et al., 2020). In addition, enforced closure of all but essential services has left many at risk of unemployment and facing economic uncertainty. Many have lost their primary source of income, which raises the unwelcome prospect of inability to afford basic costs of living including housing and food. Such threats are particularly marked among those on low incomes and underserved communities who already live paycheck to paycheck and have limited access to healthcare or benefits and face housing and food insecurity. Economic uncertainty, therefore, represents a further source of stress particularly in vulnerable groups (Van Lancker & Parolin, 2020; Yilmazkuday, 2020). Individuals employed in frontline workforces are also among those at higher risk. These workers have been directly responsible for maintaining essential services during the lockdown and have been shown to experience substantive increases in stress and vicarious traumatization (Chen et al., 2020; Law, 2020; Li et al., 2020). Previous research in similar contexts such as disasters and other traumatic events (Brackbill et al., 2006; Garfin, Thompson, & Holman, 2018; Mills, Edmondson, & Park, 2007), coupled with data from areas first affected by the virus such as China (Wang et al., 2020) and South Korea (Park & Park, 2020), have noted substantive increases in community stress levels.

The elevated stress arising from the pandemic and associated lockdown measures are likely to be prolonged even after the threat of the virus has passed. In an encouraging development, nations that have been effective in achieving declining rates of daily COVID‐19 cases, a key milestone in the goal of gaining control over the pandemic, have begun the slow, phased process of easing lockdown measures and restoring economic activities (Kupferschmidt, 2020). Businesses and public services including transportation, elective healthcare services, and educational institutions have begun to reopen, albeit with strict guidelines on social distancing and use of protective equipment where appropriate. However, the pace of the emergence from lockdown is understandably gradual, given the high extant infection rates in many areas and the omnipresent threat of a ‘second wave’ of infections (Day, 2020). This means that the financial difficulties and economic concerns remain a very real threat and will do so for a substantive period of time after the pandemic itself has passed.

The prolonged exposure to stress arising from the crisis is likely to have insidious long‐term health effects including increased risk of physical (e.g., chronic disease risk) and mental (e.g., depression, anxiety disorders and post‐traumatic stress disorder) health problems (Cohen, Janicki‐Deverts, & Miller, 2007; Kuo et al., 2019; Wu, Chan, & Ma, 2005), impaired cognitive function (McEwen & Sapolsky, 1995), and reduced productivity and absenteeism in the workplace (Kirsten, 2010). Such effects are also likely to remain long after the pandemic ends and lockdown measures lifted given that economic threats will likely persist. Chronic stress is, therefore, an important parallel public health concern during the current pandemic and in its aftermath (Garfin et al., 2020). The development of effective means to mitigate and manage stress arising from the pandemic and afterwards should, therefore, be considered a priority. It is also important that means applied to manage stress do not place increased burden on healthcare services already at or exceeding capacity (Armocida, Formenti, Ussai, Palestra, & Missoni, 2020). The onus lies on behavioural scientists to develop effective low‐cost means to assist with the management during and after the crisis.

## 2 REAPPRAISAL STRATEGIES AND STRESS MANAGEMENT IN COVID‐19

Stress‐management strategies that focus on stress reappraisal may be a promising approach. Increasing evidence suggests that individuals' beliefs about stress play an important role on their capacity to cope effectively with stress and mitigate maladaptive stress‐related outcomes (Crum, Salovey, & Achor, 2013; Jamieson, Peters, Greenwood, & Altose, 2016; Keech, Cole, Hagger, & Hamilton, 2020; Liu, Vickers, Reed, & Hadad, 2017). Many models of stress, such as the transactional model of stress and coping (Lazarus & Folkman, 1984), the biopsychosocial model of stress (Blascovich & Mendes, 2010) and the stress optimization model (Crum, Jamieson, & Akinola, 2020) suggest that stress appraisals and mindsets are central to determining whether individuals' responses to stressors are adaptive and lead to effective coping, or maladaptive and lead to ineffective coping and compromised health and functioning. A key prediction of these theories is that individuals who appraise stress as challenging, as opposed to threatening and hold beliefs that stress can be enhancing and facilitate pursuit of valued goals, as opposed to debilitating and suboptimal in goal pursuit, cope more effectively and exhibit better outcomes. These perspectives each propose two adaptive strategies that alter individuals' perspectives on stress and are likely to be highly effective in stress management: stress reappraisals and stress mindsets. Together, these strategies aim to alter the received perspective that stress is negative and leads to maladaptive outcomes including poorer health, reduced functioning, and impaired performance on tasks (A. W. Brooks, 2014; Crum, Akinola, Martin, & Fath, 2017; Crum et al., 2013; Jamieson et al., 2016; Jamieson, Mendes, Blackstock, & Schmader, 2010; Jamieson, Nock, & Mendes, 2013; Liu, Ein, Gervasio, & Vickers, 2019). Such approaches contrast with the majority of stress‐management strategies that typically aim to minimize the frequency or magnitude of felt stress and anxiety (Clough et al., 2017; Hagger & Stevenson, 2010; WHO, 2020).

Stress reappraisal interventions focus on prompting individuals to view their stress response as a resource or ‘skill’ that can be potentially beneficial (Jamieson et al., 2016; Jamieson, Mendes, & Nock, 2013). The goal of such interventions is to prompt individuals to interpret stress differently, such that higher arousal in situations once appraised as threatening and to be avoided, are instead appraised as challenging, to be approached and as facilitative of optimal performance. Such interventions are proposed to be particularly effective in contexts where the source of stress cannot be avoided and, therefore, represents a viable and adaptive alternative to strategies focused on reducing stress intensity. Stress reappraisal interventions usually involve the presentation of scenarios highlighting that stress can be effective in promoting better coping and performance, and have been shown to be effective in reducing acute stress responses and improved performance in contexts such as work and academic performance (A. W. Brooks, 2014; Jamieson et al., 2010, 2013, 2016; Jones, Hanton, & Swain, 1994; Liu et al., 2017, 2019).

A complimentary approach is offered by stress mindset theorists, who propose that individuals holding a *stress‐is‐enhancing* mindset view stress as having enhancing consequences on functioning, performance and health (Crum et al., 2013; Keech et al., 2020; Keech & Hamilton, 2020). This is contrasted with a *stress‐is‐debilitating* mindset, in which stress is viewed as having debilitating consequences on outcomes. This perspective has arisen from perspectives on implicit or ‘lay’ theories, in which individuals ‘lay’ beliefs about phenomena are characterized as either *entity* or *incremental* (Dweck, 2000). Individuals holding entity theories view phenomena like intelligence or personal qualities as fixed and unchanging, while incremental theorists view these phenomena as malleable and changeable. A *stress‐is‐enhancing* mindset is consistent with an incremental perspective, such that individuals have a flexible perspective on stress and hold beliefs that stress is an opportunity for growth with the potential to facilitate performance and functioning. In contrast, a *stress‐is‐debilitating* mindset is more consistent with an entity perspective such that individuals hold a view of stress which is more consistent with the received view that stress is harmful. A growing body of research has demonstrated that individuals endorsing a *stress‐is‐enhancing* mindset report reduced physiological stress responses, greater positive affect and cognitive flexibility, better self‐rated health, higher life satisfaction, and better academic and work performance (Casper, Sonnentag, & Tremmel, 2017; Crum et al., 2013; Keech et al., 2020; Keech, Hagger, O'Callaghan, & Hamilton, 2018). Furthermore, research in multiple contexts has demonstrated that a *stress‐is‐enhancing* mindset can be induced through intervention and have been shown to be effective in mitigating negative outcomes to highly stressful events (Crum et al., 2013, 2017; Keech, Hagger, & Hamilton, 2019).

Considering this evidence, stress reappraisal and stress mindset intervention strategies are highly efficacious, low‐cost means to assist in mitigating the deleterious effects of high levels of stress. Importantly, these strategies may be viable means to mitigate the maladaptive consequences of the considerable social upheaval and economic stress experienced during and after the pandemic and lockdown procedures, although formal evaluations of their efficacy in the context of the current COVID‐19 pandemic are needed. Such interventions may also equip those vulnerable to high stress with better capacity to cope with the easing of the lockdown and adjust more effectively to a return to work and the ‘new normal’. Stress reappraisal and mindset interventions also meet the need for a cost‐effective, low‐burden solution to pandemic‐related stress. Consistent with the broader need for cost‐effective, self‐administered behaviour change interventions (Hagger, 2010; Hagger, Cameron, Hamilton, Hankonen, & Lintunen, 2020; Hardcastle, Fortier, Blake, & Hagger, 2017; Knittle et al., 2020), stress reappraisal interventions do not require extensive client–practitioner interaction or a highly intensive administration protocol and can be self‐administered through messages and prompts delivered by remote means, such as via online, smartphone, or other devices.

## 3 HOW APPRAISAL STRATEGIES COULD ASSIST IN MANAGING STRESS DURING AND AFTER THE PANDEMIC

A number of different techniques have been adopted to administer stress reappraisal and stress mindset interventions. Research in laboratory contexts adopt minimalist approaches aimed at manipulating stress appraisals or mindsets. For example, in stress reappraisal experiments, participants are presented with text‐based messages aimed at manipulating the appraisal process (Jamieson et al., 2010, 2013; Liu et al., 2017). The messages often refer to a specific salient event (e.g., an exam or test), instruct the individual to acknowledge their feelings of stress, and present evidence that stress does not necessarily harm performance and can be facilitative. In mindset experiments, participants are presented with informational messages which advocate the enhancing nature of stress (Crum et al., 2013, 2017). Such manipulations have demonstrated the malleability of stress appraisals and mindsets and their efficacy in producing adaptive physiological, psychological, and behavioural outcomes in laboratory settings when under stress.

In the field, stress reappraisal and mindset intervention research has translated intervention materials from the lab, often in more intensive interventions that require active engagement with the content. For example, Jamieson et al. (2016) provided community college students with stress appraisal materials adapted from laboratory studies that outlined research supporting the position that stress can facilitate performance. Students receiving the reappraisal intervention reported lower math anxiety and performed better on math tests compared to those receiving a ‘placebo’ instruction. These findings are important because the intervention was conducted in a community college setting and not on high‐achieving students. Importantly, participants were required to actively acknowledge they endorsed the position advocated in the materials. Similarly, building on imagery intervention research (Conroy & Hagger, 2018; Hagger, Lonsdale, & Chatzisarantis, 2011; Hagger et al., 2012; Hamilton, Keech, Peden, & Hagger, 2019; Pham & Taylor, 1999), Keech et al. (2019) developed a novel imagery‐based technique to induce a *stress‐is‐enhancing* mindset. Participants were initially prompted to identify typical stressors in their daily life, and then engage in a series of visualization exercises in which they imagined the potentially positive consequences of stressor and the actions they could take to experience these positive consequences. The intervention was found to be effective in promoting better coping with perceived distress, adaptive changes in positive and negative affect, increased proactive behaviour and academic performance among participants with elevated stress.

These findings highlight the potential efficacy of reappraisal and mindset stress‐management strategies in mitigating both short‐ and longer‐term health outcomes and functioning. These interventions have numerous advantages. They are based on strong theory (Crum et al., 2020; Jamieson et al., 2013; Keech et al., 2018) and an evidence base developed from basic principles and in laboratory‐based research to demonstrate proof‐of‐concept (Crum et al., 2013; Jamieson et al., 2010, 2013). Importantly, they demonstrate good translatability from laboratory to field settings, with an expanding evidence base in multiple contexts demonstrating their effects with small‐to‐medium effect sizes (Jamieson et al., 2016; Keech et al., 2019). In addition, they are self‐administered and low burden, obviating time‐ and resource‐intensive in person administration, but they also require engagement with the material which ensures effortful, active attention on the part of the recipient rather than passive receipt, which may enhance their long‐term impact. Reappraisal and mindset interventions are also relatively ‘low risk’ in that they are non‐invasive and have good acceptability by participants in research adopting these techniques (Crum et al., 2020; Jamieson et al., 2013; Keech et al., 2019). However, as with many psychological approaches to stress management, there is a small risk that the process of reflecting on stress may evoke some highly traumatic stressful events in some individuals, particularly in the context of the current pandemic, which has had stressful consequences such as bereavements, loss of employment, and stressful work experiences (e.g., among healthcare and ‘frontline’ workers). Interventions that include reappraisal and mindset interventions as part of their content should, therefore, also be accompanied by information on where those who experience difficult or traumatic thoughts during the course of the intervention could seek advice and help. Taken together, these interventions stand as strong candidate means to assist with the management of stress for individuals experiencing high stress during the COVID‐19 pandemic and as nations emerge from lockdown.

Importantly, comprehensive examples of the materials for stress reappraisal and mindset interventions exist. These provide templates for adaptation to the specific contexts and populations. For example, the interactive ‘rethinking stress’ toolkits used by Crum et al. (2013) (http://sparqtools.org/rethinkingstress/) and the interactive videos, scripts, and materials used by Keech et al. (2019) (https://osf.io/3rz7n/) are available online. In practice, such interventions may be embedded in health promotion materials that are delivered to the population in print (e.g., pamphlets) and online (e.g., websites and print media) formats. For instance, the primary content of Crum et al.'s ‘rethinking stress’ toolkits are interactive videos, which challenge individuals to reconsider their view of stress and provide tips on how such reappraisals can help manage stressors that arise in the course of a typical day. Similarly, Keech et al.'s interactive videos prompt individuals to engage in imagery‐based stress mindset exercises and can be tailored to make reference to the sources of stress pertinent to the individual. These exercises could be incorporated into stress‐management interventions delivered via the Internet or mobile devices aimed at those most at risk of stress. They may also be used by clinicians and behavioural health practitioners as part of existing programs to promote stress coping. The videos and accompanying materials would need to be tailored accordingly for application to the management of stress arising from current COVID‐19 pandemic and the associated lockdown measures. This would require some minor adjustments to the content of the reappraisal and mindset intervention strategies, and identification of effective networks for administration and distribution. However, such adjustments are comparatively minor and highlight the potential flexibility of these intervention approaches to novel contexts. The high level of translatability and potential for flexible, self‐administered format suggests that stress reappraisal and mindset interventions have excellent potential to be included as part of governmental and organizational health promotion campaigns to manage stress during the current pandemic and beyond.

## 4 CONCLUSION

Stress reappraisal and mindset interventions have high potential to assist in stress management during the COVID‐19 pandemic based on their demonstrated efficacy in laboratory and selected applied contexts (Crum et al., 2013, 2017; Keech et al., 2019). Research demonstrating that such interventions are highly translatable and have consistent short‐to‐medium term effects on stress in ecologically valid contexts, suggests that it is reasonable to extrapolate previous findings to other stressful situations. However, it should be acknowledged that relatively little research has explored the effects of stress reappraisal and mindset interventions among individuals experiencing prolonged or chronic stress in the context of major stress‐inducing and traumatic events such as natural disasters or disease pandemics (Keech et al., 2020). We therefore call for research testing the efficacy of stress mindsets in mitigating stress in the context of highly stressful events, such as COVID‐19 and its aftermath, to address this evidence gap and provide definitive evidence to support their use in stress management in traumatic events such as pandemics. Such endeavours are important given that the current pandemic is likely to continue, with the potential for a ‘second wave’ and the need for greater preparedness for stress management in the event of future pandemics.

## CONFLICT OF INTEREST

The authors declare that they have no conflict of interest.
